# CRF Receptor Type 1 Modulates the Nigrostriatal Dopamine Projection and Facilitates Cognitive Flexibility after Acute and Chronic Stress

**DOI:** 10.1523/ENEURO.0019-26.2026

**Published:** 2026-03-03

**Authors:** Serena Becchi, Charlotte Lauren Burton, Madeline Tsoukalas, Jeremy Bowring, Bernard Walter Balleine, David Mor

**Affiliations:** ^1^School of Psychology, Faculty of Science, University of New South Wales, Sydney 2052, Australia; ^2^School of Medical Sciences, Faculty of Medicine and Health, University of Sydney, Sydney 2006, Australia

**Keywords:** corticotrophin-releasing factor, decision, dopamine, substantia nigra pars compacta

## Abstract

Repeated restraint stress (RRS) in rats impairs cognitive flexibility, particularly when faced with additional mild acute stress (AS). We tested the hypothesis that this impairment is associated with altered dopaminergic activity in the dorsal striatum (DS) driven by corticotropin-releasing-factor receptor type 1 (CRFR1) in the substantia nigra pars compacta (SNpc). Sixty-two male rats received RRS or handling for 14 d, before training on a two-action, two-outcome instrumental conditioning task. Initial learning was assessed using an outcome devaluation test. Cognitive flexibility was assessed by reversing the outcome identities and a second outcome devaluation test, with half the rats in each group receiving AS before reversal training. Dopamine and metabolites were quantified in the DS, and CRFR1 mRNA was quantified in the SNpc. In Experiment 2, SNpc CRFR1 was pharmacologically blocked unilaterally before AS and reversal training in 32 male and 32 female rats. Increased dopaminergic activity in the DS and SNpc and CRFR1 expression in the SNpc in the left hemisphere were associated with resilience to AS in naive rats, but with an impairment in RRS + AS rats. Blocking CRFR1 in the left SNpc impaired cognitive flexibility following AS in naive rats but restored it following AS in RRS rats. Blocking CRFR1 in the SNpc increased DA availability in the DMS but decreased it in the DLS. The study suggests opposite facilitation in DA availability in the medial and lateral DS by CRFR1 in the SNpc and a left-to-right transition in dopaminergic nigrostriatal projection activity as a protective mechanism following RRS.

## Significance Statement

This study investigated the role corticotropin-releasing-factor receptor type 1 (CRFR1) in the substantia nigra pars compacta (SNpc) in regulating cognitive flexibility following stress. Cognitive flexibility was assessed by the ability to update changes in action-outcome relationships following either chronic, acute, or a combination of both stressors. We found an increase in CRFR1 in the SNpc and in markers of dopaminergic activity in the left hemisphere of dorsal striatum associated with resilience to acute stress. In contrast, 14 d of repeated restraint plus acute stress both impaired cognitive flexibility and caused a left-to-right hemisphere transition in both CRF1 and dopaminergic activity. This transition was partly driven by opposing effects of CRFR1 on the nigrostriatal projection from the medial and lateral SNpc.

## Introduction

The dorsomedial striatum (DMS) is essential for encoding and retrieving the specific action-outcome (A-O) associations necessary for goal-directed action, both when they are initially acquired (Balleine and Dickinson, 1998; Yin et al., 2005; Peak et al., 2020) and when subsequently updated (Bradfield et al., 2013). This learning and updating depend on the modulatory influence of local dopamine (DA) release in the cortico-striatal pathway (Borwick et al., 2020; Hori et al., 2021). Stress can alter dopamine neurotransmission in the striatum in both the DMS and dorsolateral striatum (DLS). It also alters the ability of animals to adapt flexibly when A-O associations change (Baik, 2020; Yao et al., 2021; Mor et al., 2022). This has also been shown in the mesolimbic area (Mor et al., 2022) and is accompanied by shifts in the lateralization of monoaminergic activity generally (Carlson et al., 1991; Sullivan and Gratton, 1998; Sullivan and Dufresne, 2006; Molochnikov and Cohen, 2014).

Different types of stress differentially affect activity in the dorsal striatum (DS). In rodents, mild acute stress (AS) has been found to increase markers of dopaminergic activity in DMS and DLS, but only in the left hemisphere in rats showing intact cognitive flexibility (Gemikonakli et al., 2019). In contrast, chronic stress increases resting dopamine sensitivity in the substantia nigra pars compacta (SNpc) and reduces the response to rewarding stimuli, causing maladaptive striatal dopaminergic regulation (Yao et al., 2021). One of the first neuropeptides released in response to stress is corticotropin-releasing factor (CRF), which initiates humoral corticosterone release and acts centrally as a neuroregulator of cellular activity. There is a complex interaction between CRF and the midbrain dopaminergic system, with CRF input into the ventral tegmental area (VTA) facilitate rewards prediction error, but CRF input into the SNpc is found as well (Kelly and Fudge, 2018). Both CRF receptors, CRF receptor 1 (CRFR1) and CRF receptor 2 (CRFR2), are expressed in the SNpc, with CRFR1 the dominant version. CRFR1 is expressed in dopamine neurons in the SNpc that project ipsilaterally and topographically to the DS, acts as an anxiolytic and influences dopamine levels in the dorsal striatum (Korosi et al., 2006; Abuirmeileh et al., 2007, 2009; Refojo et al., 2011; Henny et al., 2012; Schneider et al., 2025). It is possible, therefore, that CRF input into the SNpc after acute and repeated stress differentially modulates both the lateralized DA response in DMS and DLS and cognitive flexibility.

To address this prediction, in the current study, we first conducted a major reassessment of brain chemistry in histological samples taken from a previous study (Mor et al., 2022) to assess changes in striatal dopamine in regions of the DS and CRFR1 mRNA expression in the SNpc in rats that received either AS, a repeated restraint stress (RRS) or both. This reanalysis suggested that CRFR1 activity in the left SNpc after AS in naive rats may function to facilitate the updating of changes in contingency but to impair it when rats were exposed to RRS prior to the AS (i.e., RRS + AS group). To establish the causal relationship between CRFR1 activation in the left or right SNpc and changes in DA markers in the DS correlated with the ability to update changes in contingencies following an AS or RRS + AS, in Experiment 2 we unilaterally infused a CRFR1 antagonist into either the medial or lateral SNpc of naive or RRS rats prior to the delivery of the AS and either reversal training or decapitation.

## Materials and Methods

### Subjects

This experiment received ethics approval from the University of New South Wales Animal Ethics Committee (AEC number 19/64B and 20/69B). The procedures adhere to the ARRIVE guidelines (Kilkenny et al., 2010). In total, 126 outbred Long–Evans rats [62 male rats in Experiment 1 and 64 (32 males and 32 females) in Experiment 2] were housed in groups of 2–4 in ventilation and humidity-controlled cages on a 12 h light/dark cycle with initial *ad libitum* access to standard chow and water.

### Experiment 1: effect of RRS and/or AS on CRFR1 expression in the SNpc, DA activity in the DS, and the consequences for behavioral flexibility

#### Apparatus

Behavioral training was conducted using 16 operant conditioning chambers (MED Associates), each enclosed within a sound- and light-attenuating shell. Each chamber contained a pellet dispenser that delivered grain pellets (45 mg, BioServe Biotechnologies) and a pump that delivered 20% sucrose solution (0.2 ml). Chambers contained two retractable levers and a recessed magazine centered between them. An infrared photobeam was positioned at the threshold of the magazine to record entries. Each chamber contained a light (3 W, 24 V) illuminated for the duration of all behavioral sessions. Sessions were preprogrammed and controlled by microcomputers running MED Associates proprietary software (Med-PC). Lever pressing, magazine entry, reinforcer delivery, and the presentation time of each lever were captured for each session using this software.

#### Repeated restraint stress

Rats were randomly assigned to naive or RRS experimental groups. The RRS treatment involved daily restraint for 2 h inside plexiglass tubes (20 cm length, 6.35 cm diameter, Ibisci, USA) over 14 d. Each day, the restraint was given at a different time between 6 A.M. and 10 P.M. to ensure the stress remained unpredictable. Rats allocated to the naive treatment were handled for a few minutes daily over the 14 d. Percentage bodyweight change was calculated at 2 d intervals during the stress period.

#### Instrumental training

Two days prior to commencing instrumental training, rats were food-restricted with daily intake restricted to 15 g of chow, which was maintained for the remainder of the experiment. Weight was monitored twice weekly to ensure it remained above 85% of baseline bodyweight.

Behavioral training started with one session of magazine training. Rats received 20 pellet and 20 sucrose outcomes at 15 s intervals for 15 min. Rats were then trained to press the levers such that, for each rat, one lever was assigned to deliver pellets and the other sucrose solution, counterbalanced to control for any lever position preference. Each training session was divided into four periods, two on each lever in alternation. During each period, one lever was extended until either 20 outcomes were delivered or 15 min had elapsed, after which the lever was retracted, and a 2 min break was instituted, after which the other lever was extended and so on. Two training sessions were conducted each day for 3 d, with the order of lever presentation counterbalanced across sessions. Outcomes were initially delivered on a continuous reinforcement schedule for three sessions, then on a random ratio (RR) 5 schedule, i.e., the outcome was delivered after five presses on average, then three sessions on RR10 and two sessions on RR20.

#### Outcome devaluation by satiety

After this training, rats completed two specific satiety-induced outcome devaluation tests on successive days. To induce specific satiety, rats were placed into clean devaluation boxes and given unrestricted access to either sucrose solution or pellets for 45 min. They were then transferred to their operant chamber to complete a 10 min extinction test. Both levers were extended simultaneously; however, no outcomes were delivered during the test. The lever associated with the outcome that was presatiated was considered the devalued lever, and the other lever was considered valued. The following day, rats received the same protocol, only with the alternative outcome presented during the prefeeding phase.

#### Mild acute stress and outcome identity reversal

To assess the rats’ ability to update changes in action-outcome (A-O) contingencies, they next received three training sessions with the outcome identities reversed; e.g., if, initially, pressing the left lever delivered a pellet and the right lever a sucrose solution, then pressing the left lever now delivered a sucrose solution and the right lever a pellet. In this phase, rats received only one training session per day, structured as previously described, with the outcomes delivered on an RR10 schedule.

During this phase, each of the previously generated stress groups was subdivided into two subgroups: one received a mild acute stress (AS), involving 5 min forced swim test, prior to each reversal training session, whereas the other received no treatment. This resulted in four groups: naive (*n* = 16), naive with AS (*n* = 13), RRS (*n* = 17), and RRS with AS (*n* = 16). For the forced swim test, rats were placed individually into a white plastic oval bin (100 cm high, 30 cm maximum diameter) filled to a height of 45 cm with clear, fresh water (at 25 ± 1°C). The subgroups under each stress condition were matched for performance in the first devaluation test to ensure similar levels of performance during initial training between the subgroups.

After reversal training, rats completed a second round of outcome devaluation tests conducted as described above, except that, after the first test, rats received a fourth refresher RR10 training session with or without the AS (as appropriate).

#### Novel mild stress and euthanasia

To assess the impact of novel AS on dopaminergic markers in the dorsal striatum and corticotropin-releasing hormone receptor 1 (CRFR1) expression in the SNpc, and to avoid the potential effects of prior experience with the forced swim test, all AS rats received a series of footshocks before killing. Rats were placed into an unfamiliar operant chamber where the stainless-steel rod floor was connected to a shock generator (Med Associates). In a single session, rats received three footshocks (5 mA) at random intervals over the course of 10 min. Rats were then transferred by hand into a novel cage where they were left for 20 min. Killing was completed by rapid decapitation without anesthesia. Brains were removed and the tissue block containing the midbrain was snap-frozen over dry ice for in situ hybridization analysis. The remaining rats were similarly killed without any additional stress treatment.

#### In situ hybridization

Frozen, unfixed tissue was sliced using a cryostat (Leica Microsystems) and mounted on Superfrost Plus slides. Ten series of 14 μm coronal sections were taken from −4.7 to −5.8 mm from bregma. Slides were stored in slide racks at −20°C for 1 h and then stored at −80°C. Identification of CRFR1 mRNA was achieved using the RNAscope 2.5HD brown reagent kit (Advanced Cell Diagnostics) and CRFR1 probe (Rn-Crhr1-C3, Advanced Cell Diagnostics). Pictures showing either the left or the right SNpc were taken using the 40× objectives. CRFR1 mRNA positive profiles in each of the stress groups were counted in the left and right, medial, and lateral SNpc at four levels (−4.9, −5.16, −5.4, and −5.64 mm from bregma) and presented as a total combined value.

#### Quantification of dopaminergic markers in the dorsal striatum using high-performance liquid chromatography (HPLC)

Dopamine (DA) and dihydroxyphenylacetic acid (DOPAC) were quantified in the left or right, DMS or DLS, of all stress groups using HPLC.

Tissue blocks containing the left or right, DMS or DLS, were microdissected under a surgical microscope. Blocks were sonicated in homogenization buffer (150 mM phosphoric acid and 500 μM diethylene triamine pentacetic acid) and centrifuged for 25 min at 16,000 rpm. An aliquot from each supernatant was used to quantify total protein using the BCA Protein Assay Kit (Thermo Fisher Scientific). The rest of the supernatant was filtered using an Amicon Ultra centrifuge filter with a cutoff of 3 kDa (Millipore). HPLC was performed using a Prominence system (Shimadzu) composed of a degasser (DGU-20A3), liquid chromatographer (LC-20AD), autosampler (SIL-20A), and communications module (CBM-20A). Isocratic mobile phase contained 13% methanol, 87% 0.01 M monobasic sodium phosphate, 0.1 mM ethylenediaminetetraacetic acid, 0.65 mM 1-octane sulfonic acid, 0.5 mM triethylamine at pH 2.81, adjusted with hydrochloric acid. Then, 7 µl of samples was injected onto a Gemini C18 column (150 × 4.60 mm, 110 Å, 5 µm particle size; Phenomenex) connected to an electrochemical detector (Antec Leyden Intro) with an Ag/AgCl reference electrode at a potential of +0.7 V and 35°C with a 1.5 ml/min flow rate. External standards for DA, DOPAC, and HVA were run daily to produce a six-point calibration curve. Peak area analysis was done using Shimadzu CLASS-VP lab solutions version 6.11 data acquisition software. Concentrations in samples were normalized to the total protein in the tissue block.

### Experiment 2: the effect of blocking CRFR1 in the SNpc on behavioral flexibility following RRS and/or AS

Experiment 2 aimed to establish whether there is a causal relationship between SNpc CRFR1 and the lateralized patterns of dopaminergic changes in the DS and cognitive flexibility following AS in naive and RRS rats. Rats were trained using a modified behavioral protocol used in Experiment 1, with the addition of a unilateral CRFR1 antagonist infusion in the SNpc prior to either the forced swim test given before the outcome reversal training or a tail-pinch stress given before decapitation.

#### Behavioral training, with CRFR1 antagonist infusion prior to AS

Following the 14 d of RRS or daily handling, all rats underwent surgery for placement of a guide cannula 1 mm above either the left or right, medial or lateral SNpc. Rats were anesthetized with isoflurane (5% induction, 2% maintenance in 100% oxygen) and positioned in a stereotaxic frame (Stoelting). They received a subcutaneous injection of Benacillin (Ilium) and Bupivacaine (Hospira) at the surgical site. An incision was made along the midline, and a small hole was drilled into the skull above the target region. A microinjection guide cannula was implanted unilaterally into either the left or right, medial or lateral SNpc. Four additional screws were fitted caudally, rostrally, and laterally to the guide cannula and secured with dental cement. Coordinates for guide cannula implantation: lateral SNpc: female rats: −5.25 mm caudal to bregma, 2.5 mm laterally from the midline, −6.6 mm below the skull; male rats: −5.3 mm caudal to bregma, 2.6 mm laterally from the midline, −7 mm below the skull, or medial SNpc: female rats: −5.35 mm caudal to bregma, 1.25 mm laterally from the midline, −7.0 mm below the skull; male rats: −5.4 mm caudal to bregma, 1.3 mm laterally from the midline, −7.4 mm below the skull.

Rats were given 7 d of recovery and monitored daily. To sustain chronic stress exposure during recovery from surgery, RRS rats received novel stress manipulations on Days 4 (2 h of wet bedding), 5 (removal of water bottles overnight), and 6 (2 h of 45° tilting of the home cage).

Rats then received magazine training, three RR1, three RR5, and three RR10 training sessions on the initial action-outcome contingencies, followed by the first set of devaluation tests. All rats then received a microinfusion of the CRFR1 antagonist antalarmin (500 ng in 250 nl of bacteriostatic 0.9% saline over 3 min; Vranjkovic et al., 2018) prior to the 5 min forced swim and outcome reversal training over the next 3 consecutive days. A second set of devaluation tests was then given over 2 consecutive days. The day following the last devaluation test, all rats received an additional antalarmin infusion, followed by a novel AS. In order to keep the AS mild and novel, the AS given was a 5 min tail pinch. Rats were then decapitated without anesthesia.

Brains were removed, and tissue blocks containing the SNpc were fixed in 4% PFA overnight, cut coronally at 40 μm using a cryostat (Leica Biosystems) and Nissl stained for confirmation of cannula location. Tissue blocks containing the left and right DMS and DLS were snap-frozen over dry ice, and DA and DOPAC levels were quantified using HPLC using the protocol described in experiment 1.

Rats with cannula placement outside the target region were excluded from the analysis. Final group numbers for the lateral SNpc infusion cohort were naive left *n* = 7, naive right *n* = 8, RRS left *n* = 8, RRS right *n* = 8, while for the medial SNpc infusion cohort were as follows: naive left *n* = 8, naive right *n* = 7, RRS left *n* = 8, RRS right *n* = 10, for a total of 64 rats in Experiment 2.

### Statistical analysis

The data were subjected to two- or three-way ANOVA or repeated-measures two- or three-way ANOVA, followed by simple effects analyses using Bonferroni’s correction for multiple comparisons when significant interactions or main effects were found. Behavioral data for both initial contingency and reversed contingency tests were also analyzed using the contrast analysis in PSY software (UNSW), which provides a more fine-grained analysis of complex experimental designs. A follow-up simple effect analysis was evaluated using PSY software.

Pearson’s correlation coefficient analysis was used to reveal correlations. The devaluation factor was calculated as the valued minus the devalued lever presses. A value of *p* < 0.05 was considered statistically significant.

## Results

### Effect of AS, RRS or their combination on behavioral flexibility

Our previous study (Mor et al., 2022) used a two-part protocol to test the effect of RRS and/or AS on behavioral flexibility, first assessing the encoding of the initial A-O identities using an outcome devaluation test before progressing to reversing the outcome identities, with half of the naive and RRS cohort also receiving an AS prior to reversal training.

All behavioral analyses, including pressing rates during original and reversal contingencies and pressing after original and reversal contingencies devaluation tests, as well as weight loss and circulating corticosterone levels following exposure to RRS, are reported in Mor et al. (2022) and in Extended Data Figure 1-1.

**Figure 1.**
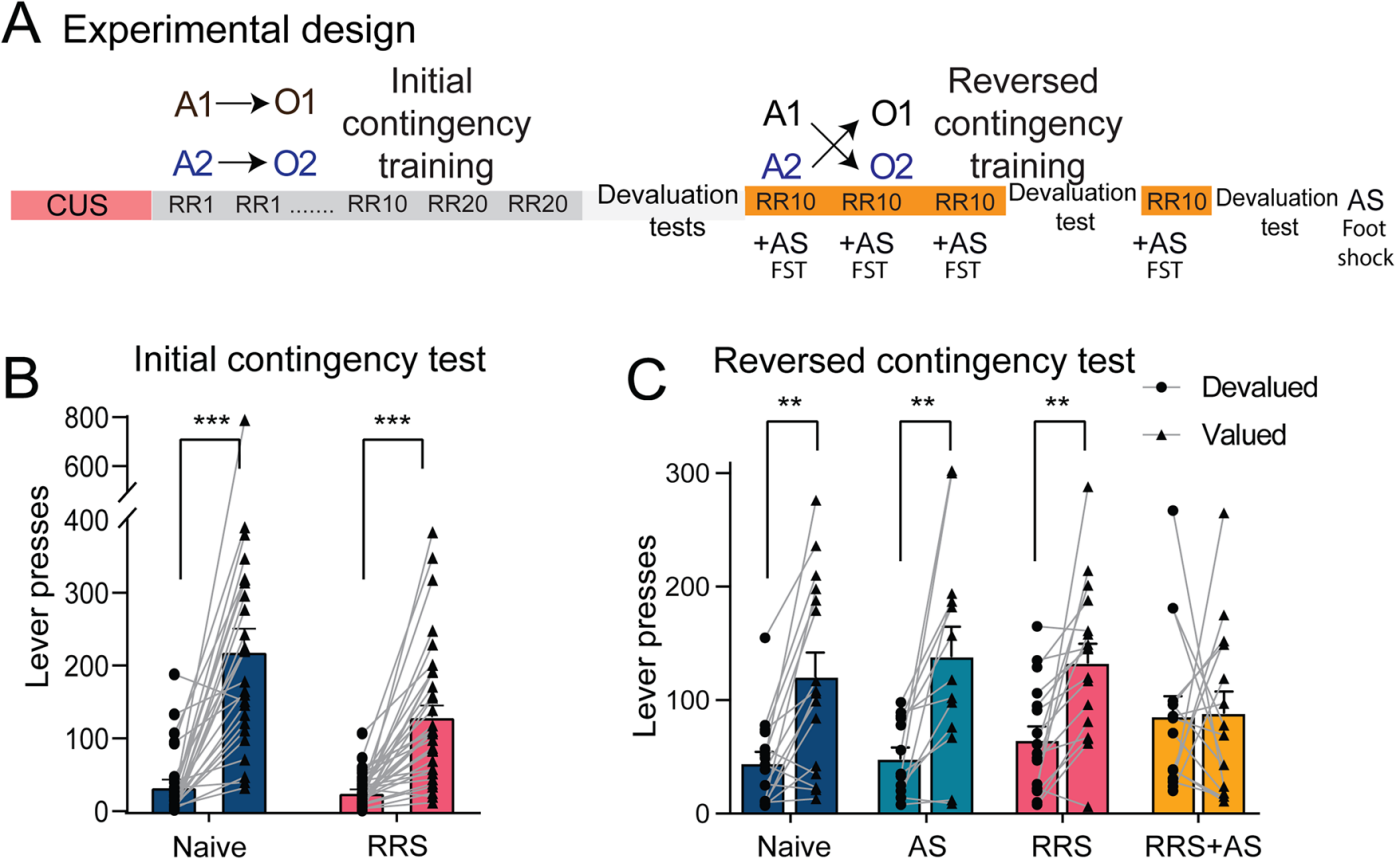
Contingency learning following chronic and acute stress exposure (adapted from Mor et al., 2022). ***A***, Design of Mor et al. (2022). ***B***, Group mean lever pressing totals over 2 d of devaluation testing for the initial contingencies. ***C***, Group mean lever pressing totals over 2 d of devaluation testing for the reversal contingencies. Error bars represent SEM. **p < 0.001, ***p < 0.0001. Extended Data Figure 1-1 supports this figure.

The experimental design is illustrated in Figure 1*A*. RRS alone did not impair the encoding of the initial A-O associations, measured using an outcome devaluation test. Two-way ANOVA showed a significant devaluation effect *F*_(1,57)_ = 73.75, with both naive and RRS groups pressing the value lever more than the devalued lever (*p* < 0.0001) and no group × devaluation interaction (Fig. 1*B*). Neither RRS nor AS alone impaired the ability to update the A-O associations after outcome identity reversal, but the combination of RRS and AS did (Fig. 1*C*). A significant devaluation effect was found in the naive (*p* < 0.001), AS (*p* < 0.001) and RRS (*p* < 0.003) groups but not in the RRS + AS group (*p* = 0.901). A 2 × 4 mixed ANOVA also revealed a significant overall effect of outcome devaluation, *F*_(1,55)_ = 27.81, *p* < 0.001 and a devaluation × group interaction *F*_(3,55)_ = 3.10, *p* = 0.034.

10.1523/ENEURO.0019-26.2026.f1-1 Figure 1-1 **Contingency Learning Following Chronic and Acute Stress Exposure (adapted from Mor et al., 2022) A**) Group mean lever pressing rates during initial learning. A repeated measures ANOVA showed pressing rates increased over the training, F_(11,52)_ = 30.79, *p* < .001, but that this increase differed between the CUS and Naïve rats, yielding a stress × training interaction, F_(11,52)_ = 2.29, *p* = .023. Pressing rates were significantly lower for CUS than Naïve rats, F_(1,62)_ = 6.75, *p* = .012, suggesting attenuated rigour after CUS. **B**) Group mean lever pressing rates during reversal learning. A repeated measures ANOVA revealed that pressing rates increased significantly over sessions, *F*_(3,165)_ = 15.96, *p* < .001, and there was no interaction between group and sessions, *F*_(9,165)_ = 1.81, *p* = .070. There was, however, evidence of a difference in lever pressing between treatment groups *F*_(3,55)_ = 6.55, *p* < .001, with main effects for CUS *F*_(1,55)_ = 12.38, *p* < .001 as well as AS *F*_(1,55)_ = 7.42, *p* = .008. Bonferroni adjusted pairwise comparisons indicated reduced pressing rates in CUS with AS when compared with Naïve rats on training days one (*p* = .009), two (*p* = .002) and four (*p* = .012). Bars represent means ± SEM. *p < .05, **p < .01, ***p < .001. Download Figure 1-1, TIF file.

### Effect of AS, RRS, or their combination on DA and DOPAC levels in DS

**Figure 2.**
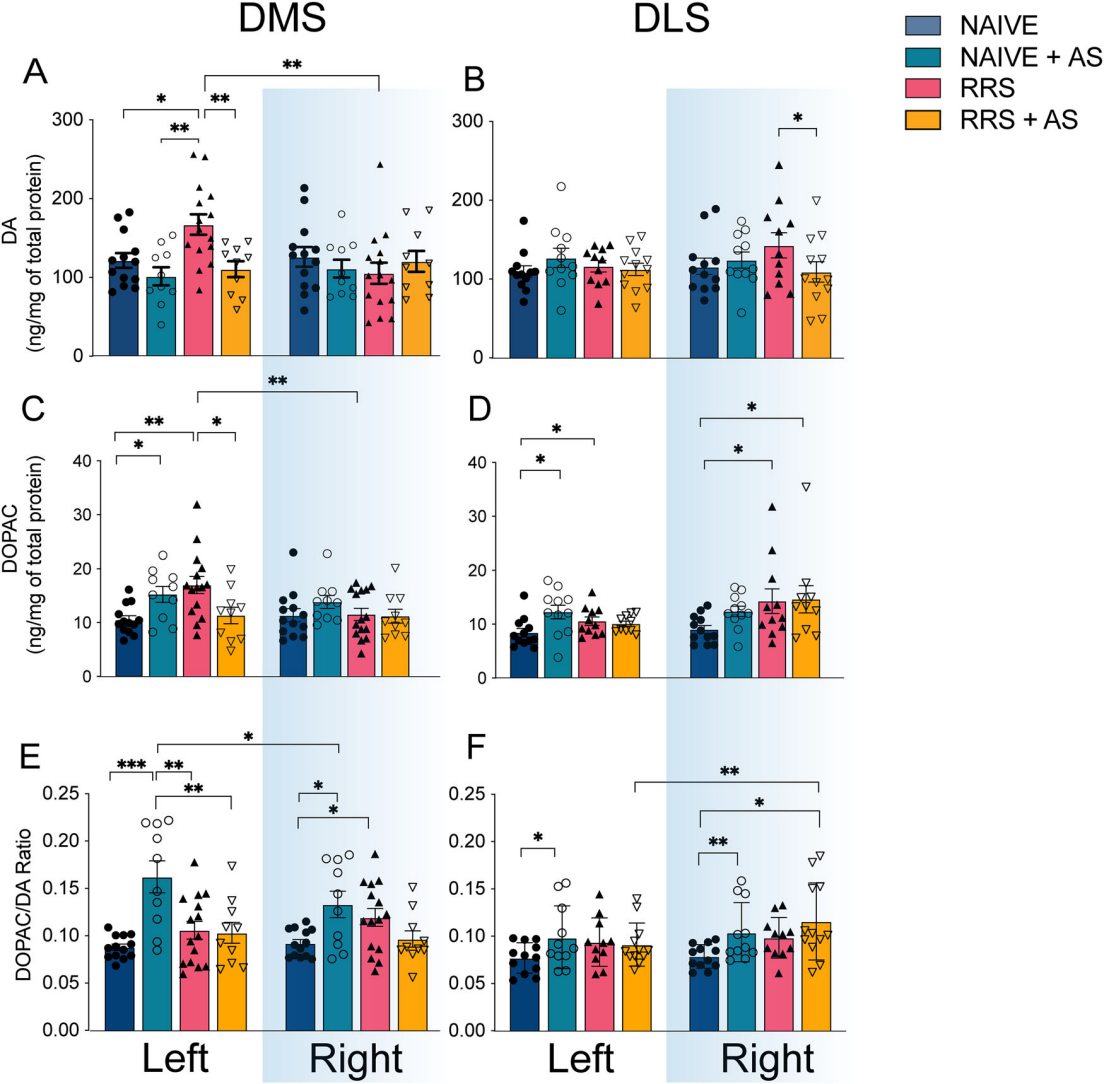
The effect of AS, RRS, or the combination of both on DA and DOPAC in the DS. ***A***, ***C***, ***E***, Quantification of DA (***A***), DOPAC (***C***), and DOPAC/DA ratio (***E***) in the left and right hemispheres in the DMS of rats that received 2 weeks of RRS or handling with or without AS for 3 consecutive days just before reversal training. ***B***, ***D***, ***F***, Quantification of DA (***B***), DOPAC (***D***), and DOPAC/DA ratio (***F***) in the left and right hemispheres in the DLS of the same rats. Error bars represent means ± SEM. Naive *n* = 16, naive + AS *n* = 13, RRS *n* = 17, RRS + AS *n* = 16. *p < 0.05, **p < 0.01, ***p < 0.001. Extended Data Figure 2-1 supports this figure.

Changes in DA and DOPAC levels in the left and right, DMS and DLS, following either AS, RRS or both, were assessed at the end of the behavioral task using HPLC (Fig. 2). A detailed summary of all interactions, main effects, and post hoc analyses is available in Extended Data Figure 2-1.

10.1523/ENEURO.0019-26.2026.f2-1 Figure 2-1 **Statistical summary table.** Tests of within-subjects contrasts and between-subject effects for DA, DOPAC and the DOPAC/DA ratios in DMS and DLS tissue blocks from experiment 1. Download Figure 2-1, DOCX file.

In the DMS, both RRS and AS increased dopaminergic markers selectively in the left hemisphere. RRS increased DA levels (*p* = 0.004; Fig. 2*A*), and both RRS (*p* = 0.001) and AS (*p* < 0.05) increased DOPAC levels (Fig. 2*C*). No increases in either DA or DOPAC were found on the right following either RRS or AS. The DOPAC/DA ratio, which is indicative of DA turnover, increased bilaterally following AS alone (left: *p* < 0.001, right: *p* = 0.001; Fig. 2*E*), with a larger increase in the left compared with the right (*p* = 0.01). When combined, RRS and AS reversed the increases in DA (*p* = 0.001; Fig. 2*A*) and DOPAC (*p* = 0.007; Fig. 2*C*) levels as compared with RRS alone.

The differential effect of the stressors and the selective effect found in the left hemisphere resulted in three-way AS × RRS × hemisphere interactions for DA: *F*_(1,44)_ = 4.090, *p* = 0.049 and DOPAC: *F*_(1,44)_ = 5.173, *p* = 0.028, and an AS × RRS interaction for the DOPAC/DA ratio *F*_(1,41)_ = 20.534, *p* < 0.001.

In the DLS, AS increased DOPAC levels (*p* = 0.008; Fig. 2*D*) selectively in the left hemisphere and the DOPAC/DA ratios bilaterally (left *p* = 0.045, right *p* = 0.005; Fig. 2*F*), while RRS alone increased DOPAC levels bilaterally (left *p* = 0.023, right *p* = 0.004; Fig. 2*D*). The combined RRS and AS lead to selective changes in the right hemisphere with reduced DA levels when compared with RRS alone (*p* = 0.01; Fig. 2*B*), increased DOPAC levels when compared with naive rats (*p* = 0.011; Fig. 2*D*), and higher DOPAC/DA ratios on the right when compared with the left of the same rats (*p* = 0.014; Fig. 2*F*). The differential effects of the different stresses lead to RRS × AS interactions for DA, *F*_(1,40)_ = 8.124, *p* = 0.007 and DOPAC *F*_(1,40)_ = 14.942, *p* < 0.001.

**Figure 3.**
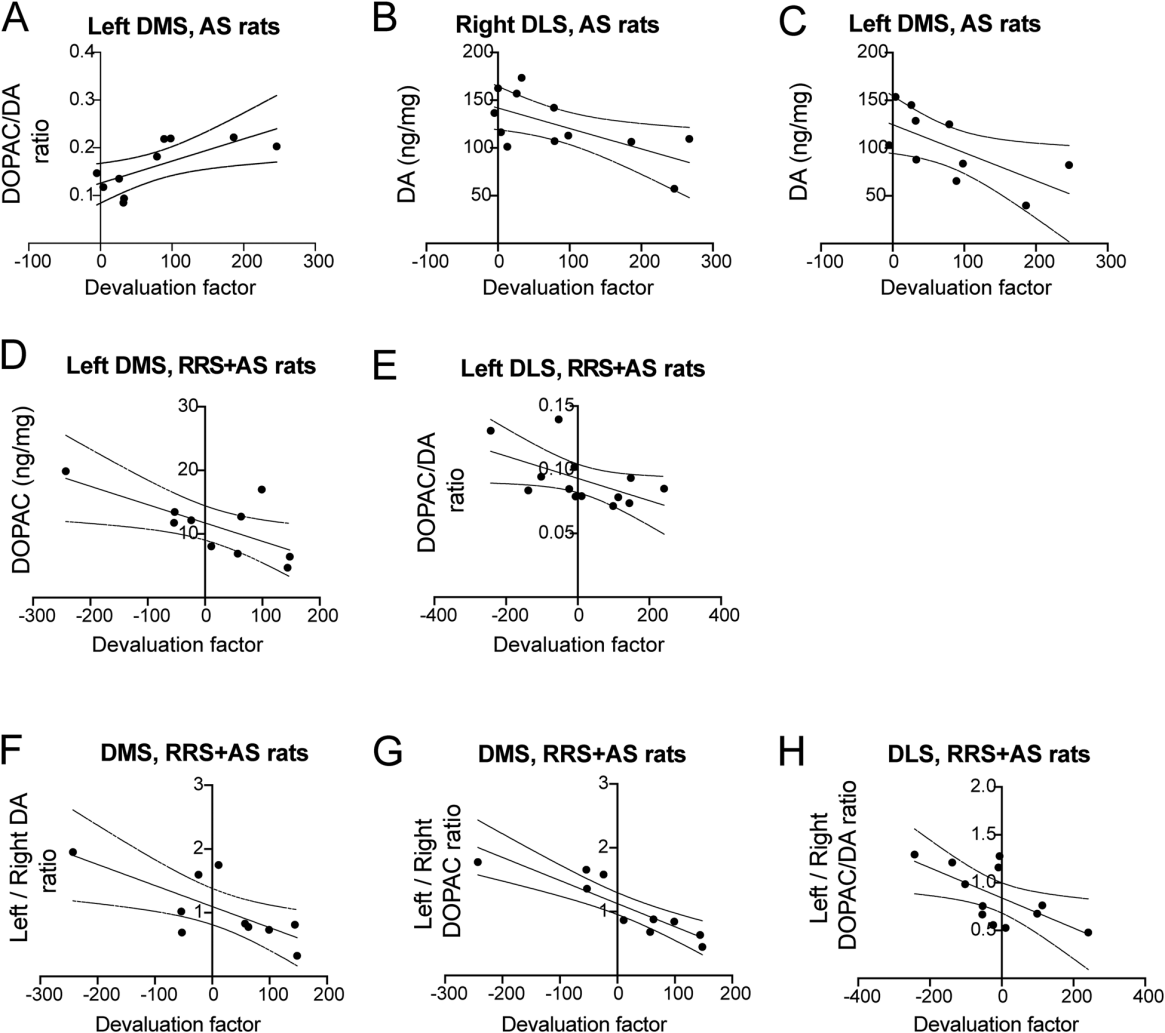
Correlations between monoamine levels and behavioral performance at test. ***A–C***, Pearson’s correlations between the devaluation factor in AS rats and the DOPAC/DA ratios in the left DMS (***A***), DA level in the right DLS (***B***), and DA level in the left DMS (***C***). ***D***, ***E***, Correlation between the devaluation factor in RRS + AS rats and the DOPAC levels in the left DMS (***D***) and DOPAC/DA ratios in the left DLS (***E***). ***F–H***, Correlations between the devaluation factor in RRS + AS rats and the left/right ratios of DA in DMS (***F***), DOPAC in the DMS (***G***), and DOPAC/DA in the DLS (***H***). Extended Data Figure 3-1 supports this figure.

To identify relationships between individual differences in performance and learning with dopaminergic activity in the DS, we calculated the correlation between DA, DOPAC, and the DOPAC/DA ratios and lever-press rates during reversal learning sessions (Extended Data Fig. 3-1) and performance at test after reversal training, represented by the devaluation factor, calculated as presses on the valued lever minus presses on the devalued lever. Significant correlations between devaluation factors and DA, DOPAC, and the DOPAC/DA ratios were found only in groups receiving AS, either AS alone (Fig. 3*A–C*) or in combination with RRS (Fig. 3*D*,*E*).

10.1523/ENEURO.0019-26.2026.f3-1 Figure 3-1 **Correlations between monoamine levels and behavioral performance during reversal training.** During reversal training, pressing rates in both naïve and AS rats decreased with increased dopaminergic activity in the right DLS, with negative correlations with DOPAC in naïve (r^2^=.627, *p* = .006) (**A**) and DA in AS rats (r^2^=.411, *p* = .024) (**B**). In the left DMS, AS and CUS led to opposite associations, with pressing increasing in AS with increased DOPAC/DA ratios (r^2^=.441, *p* = .0360) (**C**) and decreasing with increased DA in CUS rats (r^2^=.313, *p* = .037) (**D**). In contrast to what was found in the left DMS though, CUS rats pressing increased with increased DOPAC/DA in the left DLS (r^2^=.355, *p* = .04) (**E**). Download Figure 3-1, TIF file.

Following an AS alone, the ability of the rat to encode changes in A-O associations improved with increased DOPAC/DA ratio in the left DMS (*r*^2^ = 0.5, *p* = 0.022; Fig. 3*A*) and decreased DA levels in right DLS (*r*^2^ = 0.411, *p* = 0.024; Fig. 3*B*) and left DMS (*r*^2^ = 0.433, *p* = 0.038; Fig. 3*C*). Following the combined AS and RRS, the ability of the rat to encode changes in A-O associations decreased with increased dopaminergic activity in the left DS, with negative correlations found with DOPAC levels in the left DMS (*r*^2^ = 0.697, *p* = 0.025; Fig. 3*D*) and with DOPAC/DA in the left DLS (*r*^2^ = 0.308, *p* = 0.049; Fig. 3*E*).

Finally, to test whether the magnitude of the asymmetry between the two hemispheres played a role in updating A-O associations, correlations were assessed between devaluation factors and the ratio of monoamine levels in the left and right hemispheres of the DMS or DLS. Significant relationships were found only following the combined RRS + AS, with negative correlations found between performance and increasing left/right ratios in DA (*r*^2^ = 0.528, *p* = 0.017; Fig. 3*F*) and DOPAC (*r*^2^ = 0.787, *p* < 0.00; Fig. 3*G*) in the DMS and DOPAC/DA in the DLS (*r*^2^ = 0.417, *p* = 0.023; Fig. 3*H*), indicating reduced performance when left measures were higher than the right and improved performance when measures on the right were higher than the left.

### The effect of AS, RRS, or their combination on CRFR1 mRNA expression in the SNpc

**Figure 4.**
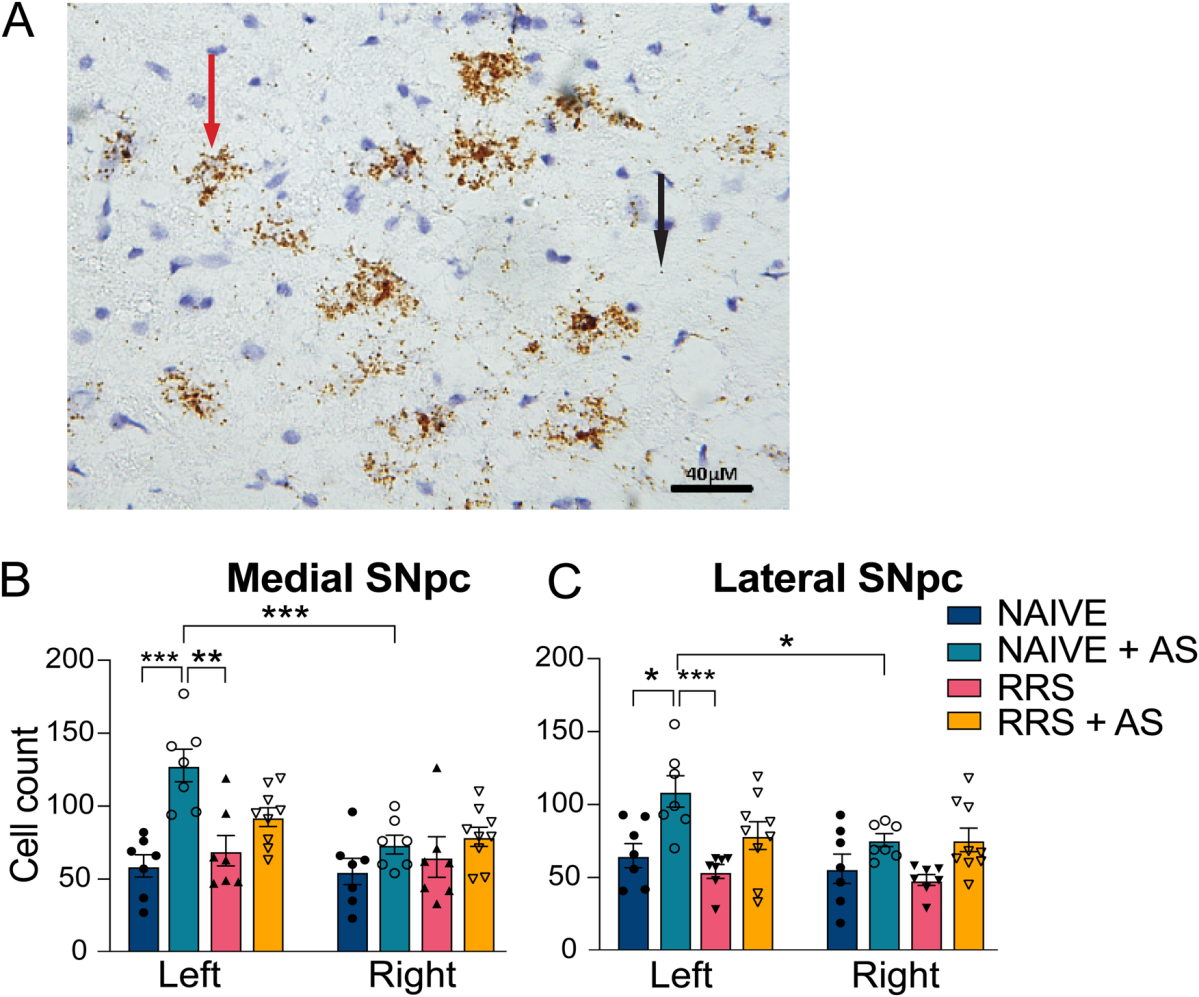
Quantification of CRFR1 in the SNpc with in situ hybridization. ***A***, Micrographs representing the staining of CRFR1-positive cells using in situ hybridization. Staining revealed clusters of mRNAs (red arrow) or a single mRNA (black arrow). Clusters of signals were counted and reported for both the medial SNpc (***B***) and the lateral SNpc (***C***) for all four groups in both the left and right hemispheres. Error bars represent means ± SEM. *p < 0.05, **p < 0.01, ***p < 0.001.

CRFR1 mRNA expression in the left and right, medial and lateral SNpc was quantified using in situ hybridization. Staining allowed the identification of individual mRNA molecules, and a cluster of mRNA was considered a positively stained cell (Fig. 4*A*).

AS alone increased CRFR1-positive cells in both the left medial (*p* < 0.001) and lateral (*p* < 0.05) SNpc (Fig. 4*B*,*C*). No increases following AS alone were found in the right SNpc or when the AS was given following RRS. The selective increases on the left SNpc resulted in significantly higher numbers in the left in comparison with the right in both the medial (*p* < 0.001) and lateral (*p* = 0.035) SNpc. This pattern resulted in a side × AS × RRS three-way interaction *F*_(1,26)_ = 6.674, *p* = 0.015 and a side × AS interaction *F*_(1,26)_ = 11.15, *p* = 0.002 in the medial SNpc, main effects for AS in both the medial *F*_(1,26)_ = 17.67, *p* < 0.001 and lateral *F*_(1,26)_ = 18.97, *p* < 0.001 SNpc, and a main effect for side in the medial *F*_(1,26)_ = 21.47, *p* < 0.001 and the lateral *F*_(1,26)_ = 7.407, *p* = 0.011 SNpc.

### Experiment 2: the effect of lateralized CRFR1 antagonist infusions on cognitive flexibility

HPLC and in situ data from Experiment 1 suggested a lateralized effect of CRFR1 activation in the SNpc and dopaminergic activity in the DS on the ability to update changes in A-O associations following AS. The data suggest that CRFR1 activity in the left SNpc facilitated the acquisition of new contingencies after AS in the naive group but impaired it in rats that had received RRS (i.e., RRS + AS group). To test this hypothesis, we unilaterally infused the CRFR1 antagonist, antalarmin, in the medial or the lateral SNpc prior to the AS and reversal training (see Extended Data Fig. 5-1 for placements).

Rats received 14 d of RRS or daily handling, during which the naive group gained significantly more body weight than the RRS group (Extended Data Fig. 5-1*B*,*C*). Subsequently, all rats were food-restricted and given training on the two-lever two-outcome task (pressing rates presented in Extended Data Fig. 5-1*D*,*E*).

**Figure 5.**
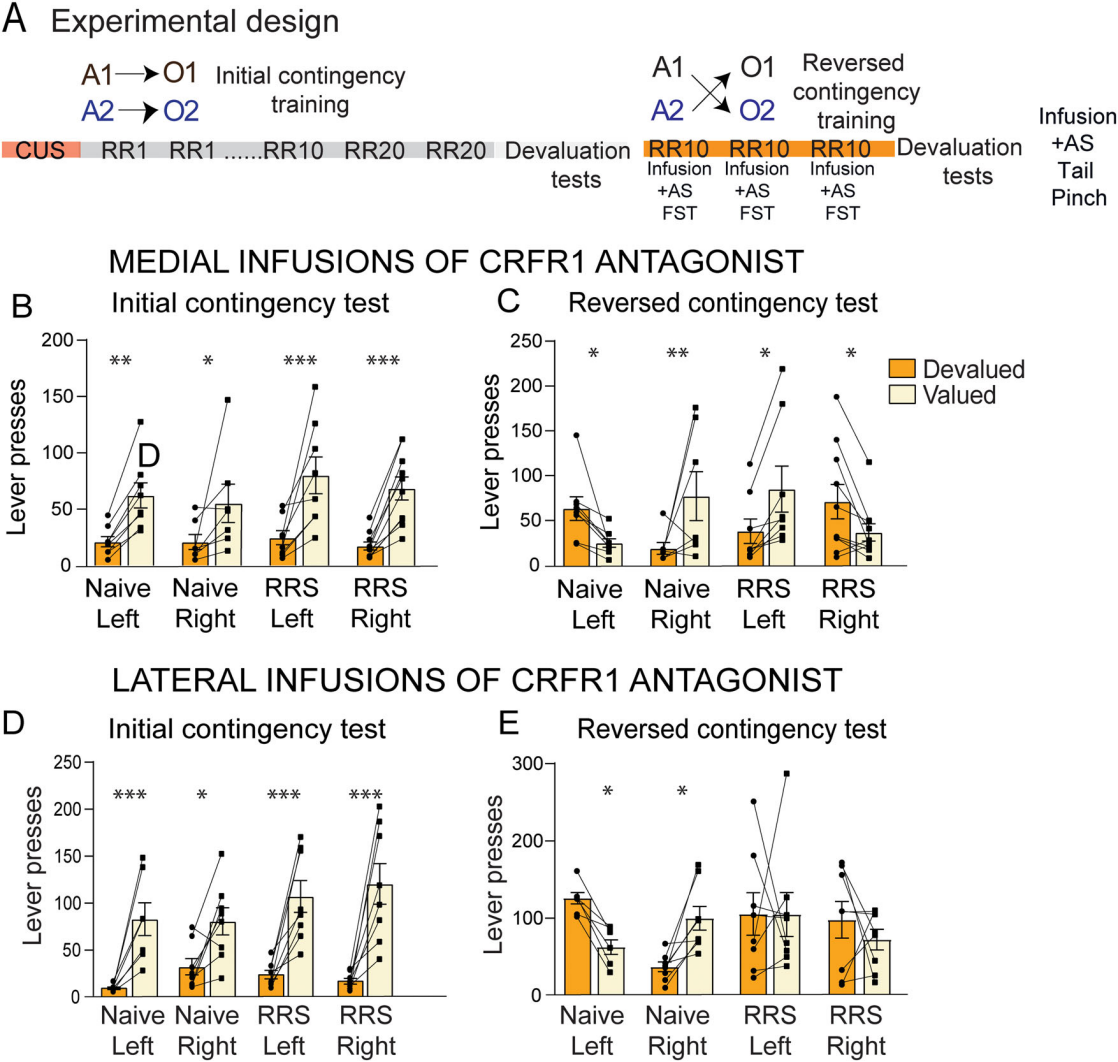
The effect of lateralized CRFR1 antagonist infusions on cognitive flexibility. ***A***, Experiment design for Experiment 2. ***B***, ***D***, Group mean lever pressing totals over 2 d of devaluation testing for the initial contingencies in rats that received unilateral cannula implantation in the left or right medial (***B***) and lateral (***D***) SNpc. ***C***, ***E***, Group mean lever presses during outcome devaluation test following outcome identity reversal training in rats that received a unilateral infusion of CRFR1 antagonist into either the left or right medial (***C***) or lateral (***E***) SNpc and an AS before each training session. Medial cohort: naive left *n* = 8, naive right *n* = 7, RRS left *n* = 8, RRS right *n* = 10, lateral cohort: naive left *n* = 7, naive right *n* = 8, RRS left *n* = 8, RRS right *n* = 8. Error bars represent means ± SEM. *p < 0.05, **p < 0.01, ***p < 0.001. Extended Data Figure 5-1 supports this figure.

A-O encoding was confirmed using a sensory-specific satiety-induced outcome devaluation test. Three-way ANOVA revealed a significant devaluation effect in both medial SNpc and lateral SNpc cannula cohorts: *F*_(1,27)_ = 73.885, *p* < 0.001 (lateral), and *F*_(1,29)_ = 57.86, *p* < 0.001 (medial), with no significant interactions in either cohort. A significant devaluation effect was found in all of the groups, suggesting similar encoding of A-O associations during training (Fig. 5*A*,*C*).

10.1523/ENEURO.0019-26.2026.f5-1 Figure 5-1 **The effect of lateralized CRFR1 antagonist infusions on body weight and instrumental training. A**) Map of cannula placements for lateral and medial SNpc cohorts. Percentage bodyweight for the lateral cannula cohort (**B**) and the medial cannula cohort (**C**) across 2 weeks of CUS or handling. Bodyweight was collected every second day. 2 × 7 ANOVA revealed a significant time x stress interaction, in both the lateral SNpc cohort: F(_6, 162_) = 8.892, p < .001 and the medial SNpc cohort : F(_6, 174_) = 13.99, p < .001. **D-E**) Lever press training for lateral (D) and medial (E) cannula animals during the initial and reversed contingency training. Throughout training, both medial and lateral cannula cohorts quickly acquired lever contingencies, as pressing rates increased over time. During initial contingency training they showed an overall effect of training F_(8, 232)_ = 69.04, *p* < .001 and F_(2.542, 68.64)_ = 78.10, *p* < .0001, respectively, with no significant differences between group Naïve and CUS (all Fs < 1) or implantation location, all Fs < 1, *p* > .05. During training on the reversed contingency, a mixed 2 × 2x3 ANOVA revealed a significant main effect as pressing rate increased over sessions: medial cohort *F*_(2, 58)_ = 7.963, *p* < .001, lateral cohort *F*_(1.778, 48)_ = 23.39, *p* < .0001. No interactions between session x stressor was found for either cohort: medial infusion *F*_(2, 58)_ = 1.228, *p* > .05, lateral infusions *F*_(2, 54)_ = 1.063, *p* > .05, neither any interaction between session and infusion side (left or right): medial infusion *F*_(2, 58)_ = 1.052, *p* > .05, lateral infusions *F*_(2, 54)_ = 2.928, *p* = .062. Bars represent means ± SEM. *** *p* < .001. Download Figure 5-1, TIF file.

Next, all rats received outcome identity reversal training, together with an infusion of antalarmin and AS before each training session (pressing rates presented in Extended Data Fig. 5-1*D*,*E*), followed by a second outcome devaluation test.

All four groups that received medial SNpc infusions had a significant difference between presses on the valued and devalued levers (Fig. 5*B*), though the AS and the RRS + AS groups showed opposite patterns of pressing between left and right infusions. AS rats pressed more on the valued lever following a right-side infusion (AS Right × devaluation *F*_(1,29)_ = 9.871, *p* = 0.004), indicating correct updating of A-O identities, but more on the devalued lever following a left-side infusion (AS left × devaluation *F*_(1,29)_ = 4.922, *p* = 0.0345), indicating performance according to the initial contingency. This opposite pattern between left and right infusions resulted in a side × devaluation interaction (AS left vs AS eight × devaluation *F*_(1,29)_ = 14.516, *p* < 0.001). An opposite pattern between left and right infusions was also found in RRS + AS rats, but, in these groups, left-side infusion resulted in higher pressing on the valued lever (RRS + AS Left × devaluation *F*_(1,29)_ = 7.336, *p* = 0.011), indicating correct updating of A-O identities, whereas right-side infusion resulted in higher pressing on the devalued lever (RRS + AS right × devaluation *F*_(1,29)_ = 4.884, *p* = 0.035), indicating performance according to the initial contingencies, again resulting in a side × devaluation interaction (*F*_(1,29)_ = 12.194, *p* = 0.002). Finally, the opposite patterns of left and right infusions between the AS and RRS + AS groups resulted in a significant three-way devaluation × stress × side interactions (*F*_(1,29)_ = 26.71, *p* < 0.001).

In rats given lateral SNpc infusions, only the AS groups showed a significant devaluation effect (Fig. 5*D*), and, in a similar manner to that seen following medial SNpc infusions, an opposite pattern was observed between the left and right infusions. Following a right-side infusion, pressing was higher on the valued lever (AS right × devaluation *F*_(1,27)_ = 5.007, *p* = 0.003), indicating correct updating of A-O identities, whereas, following a left-side infusion, pressing was higher on the devalued lever (AS left × devaluation *F*_(1,27)_ = 4.440, *p* = 0.045), indicating rats were still performing according to the initial contingency. This opposite pattern between left and right infusions resulted in a side × devaluation interaction (*F*_(1,27)_ = 9.409, *p* = 0.005). No devaluation effects or side × devaluation interaction were found in the RRS + AS groups. Nevertheless, the different patterns between the AS and RRS + AS groups resulted in a significant three-way devaluation × stress × side interaction (*F*_(1,27)_ = 6.991, *p* = 0.014).

### Effect of antalarmin infusion on DA and DOPAC levels in DS following AS in naive and RRS rats

To assess the influence of novel stress on the relationship between SNpc CRFR1 and DA and DOPAC levels in the DS, 30 min prior to decapitation, all rats received a unilaterally infused antalarmin in the SNpc and a 5 min tail pinch. DA and DOPAC levels were quantified in DMS and DLS tissue blocks from the infused and noninfused hemispheres. A detailed summary of all interactions, main effects and post hoc analyses is available in Extended Data Figure 6-1.

**Figure 6.**
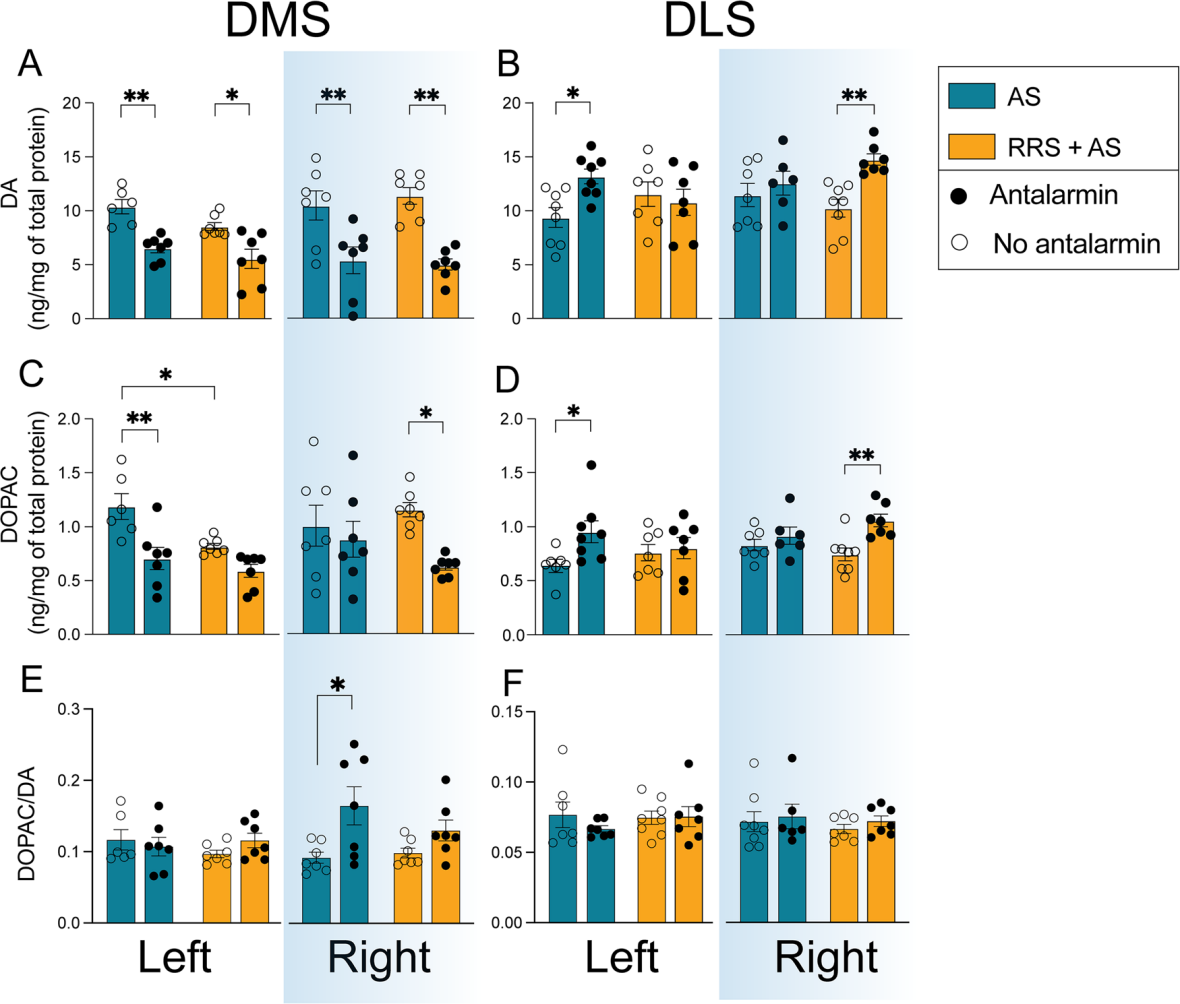
The effect of SNpc antalarmin infusion on DA and DOPAC levels and the DOPAC/DA ratios in the DS following AS in naive and RRS rats. ***A***, ***C***, ***E***, Quantification of DA (***A***), DOPAC (***C***), and DOPAC/DA ratios (***E***) in the left and right DMS of rats that received 2 weeks of RRS or handling combined with and without a medial SNpc antalarmin infusion prior to an AS. ***B***, ***D***, ***F***, Quantification of DA (***B***), DOPAC (***D***), and DOPAC/DA (***F***) in the left and right DLS that received 2 weeks of RRS or handling combined with and without a lateral SNpc antalarmin infusion prior to an AS. Error bars represent means ± SEM. *n* = 7 per group. *p < 0.05, **p < 0.01. Extended Data Figure 6-1 supports this figure.

Blocking CRFR1 in either the left or right medial SNpc reduced DA levels in the left and right DMS, respectively, in both the AS and RRS + AS groups (infused vs noninfused: AS rats *p* < 0.01 in both left and right DMS, RRS + AS rats left *p* < 0.05, right: *p* < 0.01; Fig. 6*A*), resulting in a main effect of infusion on both sides: left: *F*_(1,23)_ = 30.14, *p* < 0.001 and right: *F*_(1,24)_ = 30.89, *p* < 0.001. In contrast, the effect of the infusion on DOPAC levels was lateralized, with reduced levels in AS group found only in the left DMS (*p* < 0.01) and in RRS + AS rats in the right DMS (*p* < 0.05; Fig. 6*B*), resulting in a main effect of infusion: left: *F*_(1,23)_ = 18.48, *p* < 0.001 and right: *F*_(1,24)_ = 6.165, *p* < 0.05.

10.1523/ENEURO.0019-26.2026.f6-1 Figure 6-1 **Statistical summary table.** Tests of within-subjects contrasts and between-subject effects for DA and DOPAC in the DMS and DLS tissue blocks from Experiment 2. Download Figure 6-1, DOCX file.

Reduced DOPAC levels in the noninfused left DMS of RRS + AS rats, when compared with the noninfused left DMS of AS rats (*p* < 0.05; Fig. 6*B*), suggested reduced levels of stress-induced DA increase in the left DMS independently of the infusion, resulting in a main effect of RRS, *F*_(1,23)_ = 8.62, *p* < 0.01.

Antalarmin infusion into the lateral SNpc prior to AS had the opposite effect on DA and DOPAC levels in the DLS to that observed in the DMS following the medial SNpc infusion. Following infusion and AS, DA and DOPAC levels were increased in the left DLS in AS rats (*p* < 0.05) and the right DLS of RRS + AS rats (*p* < 0.01; Fig. 6*B*,*D*). This resulted in a RRS × infusion interaction for DA in the left DLS, *F*_(1,26)_ = 5.374, *p* = 0.028, and a main effect of infusion on DA levels in the right DLS, *F*_(1,24)_ = 9.657, *p* < 0.001, and on DOPAC levels bilaterally: left, *F*_(1,25)_ = 4.854, *p* = 0.037, and right, *F*_(1,24)_ = 10.36, *p* < 0.01.

Due to reduced DA levels, but no change in DOPAC, a main effect for infusion on DA turnover was found in the right, *F*_(1,24)_ = 10.49, *p* = 0.003, with increased turnover rate in the right DMS of AS rats (Fig. 6*E*).

## Discussion

We have previously shown that a mild AS or RRS alone do not impair learning after outcome identity reversal, but their combination does. Here, we show that these differences are associated with opposing changes in lateralized DA activity in the DS. Increased DA activity in the left hemisphere promotes learning following AS or RRS. However, following exposure to both stressors, a transition in DA activity from left to right hemisphere may allow resilience from further stress. Furthermore, these lateralized differences were driven by CRFR1 in the SNpc, which facilitated an increase in DA level in the DMS but reduced it in the DLS following stress.

### Asymmetries in DS DA activity induced by AS in naive and RRS rats

An endogenous asymmetry in dopaminergic activity within the DS has been reported previously in humans and rodents (Molochnikov and Cohen, 2014). This is often associated with motor functions (Zimmerberg et al., 1974; Glick et al., 1988; Mor et al., 2022), as well as the strength of the action-outcome association independently of changes in movement (Hart et al., 2024). We showed that following stress, three different patterns of dopaminergic markers asymmetry emerged in the striatum. AS increased dopaminergic activity in the left DMS and DLS, with positive correlations between learning score and DA metabolism in the left DMS. This is consistent with other studies showing preferential changes in dopaminergic activity markers following mild stress in the left DS (Gemikonakli et al., 2019), left cortical regions associated with behavioral selection (Carlson et al., 1991), and increased c-fos in the left DS following a forced swim test (Campus et al., 2017).

RRS also increased DA and DOPAC in the left DMS, but in the DLS, these increases were larger in the right hemisphere and were associated with reduced, but still intact, learning. Finally, when RRS rats were given additional AS, increased DA turnover in the right DLS remained, whereas levels in the left DMS were reduced. These patterns suggest a possible transition in DA activity from left to right hemispheres with increasing severity of stress. Whereas AS rats showed positive correlations between learning and DA turnover, rats with RRS + AS showed negative correlations, with improved learning associated with reduced turnover in left DMS and DLS. This suggests that the left-to-right transition may be a protective mechanism and a marker of resilience. Furthermore, uniquely to RRS + AS rats, the magnitude of the asymmetry, rather than the concentration on each side individually, appeared to play a role in the rats’ capacity to retain learning after AS: rats with lower left versus right ratios in both the DMS and DLS showed better performance. Increased dopaminergic transmission within the dorsal striatum can also result from CRFR1 activation of local cholinergic interneurons (Lemos et al., 2019), which are essential for updating action-outcome contingencies and learning related plasticity (Bradfield et al., 2013). It will also be important, therefore, to establish whether there is an asymmetry in CRF modulation of cholinergic interneurons following acute and/or chronic stress and its influence on cognitive flexibility. However, whether these reflect asymmetries in dopamine activity and striatal output remains an open question. An important next step will be measuring extracellular DA release in the striatum following stress and identifying whether this release primarily influences activity in D1 or D2-containing neurons.

Caution should also be exercised in interpreting changes in DA levels, as most of the DA quantified reflects presynaptic storage, whereas levels of DOPAC or DOPAC/DA indicate DA metabolism and potentially activity. This could explain the opposite patterns found in DA and DOPAC/DA in the left DMS following AS. Finally, since AS and reversal training last for >80 min, it is important to recognize that the infusion made into the lateral or medial SNpc could have diffused beyond the boundaries of the injected regions and so may have influenced neighboring regions.

### Asymmetries in SNpc CRFR1 expression induced by AS in naive and RRS rats

Assessments of CRF modulation of midbrain dopaminergic responses to stress have focused almost exclusively on the VTA and its projections. Maladaptations of the CRFR1 and CRFR2 in the VTA are associated with substance dependence, stress-induced relapse, and the motivational drive of decision-making (Beckstead et al., 2009; Lemos et al., 2012; Mor et al., 2022; Zalachoras et al., 2022).

Neurons in the SNpc also express CRFR1 (Sauvage and Steckler, 2001) and have substantial CRF input from key regions regulating behavioral responses to stress (Kelly and Fudge, 2018). Activation of CRFR1 in the SNpc modulates the firing of dopaminergic neurons by enhancing the inhibitory postsynaptic current, a process that was attenuated by a week of repeated daily restraint (Beckstead et al., 2009). AS in naive rats increased CRFR1 expression in the left medial and lateral SNpc, a process which was attenuated in RRS + AS rats. To confirm whether activation of CRFR1 produced the described DA changes in the DS and the subsequent differences in cognitive flexibility, in Experiment 2, we blocked CRFR1 during AS before outcome identity reversal training. Since neurons in the SNpc topographically project to the DS, we infused CRFR1 antagonist either in the lateral or medial SNpc, therefore affecting the DLS or the DMS DA release, respectively. Blocking CRFR1 in the left medial and lateral SNpc following AS impaired learning during reversal training. These rats performed according to the original contingencies in the reversal devaluation test. In contrast, blocking CRFR1 in the right medial or lateral SNpc did not impair this learning and rats performed according to the reversed associations. These findings further support those of Experiment 1 and suggest that CRFR1-induced dopaminergic activity in both the left DMS and DLS is required for cognitive flexibility following stress.

In contrast, blockade of CRFR1 in the left medial SNpc of RRS + AS rats appeared to mitigate the learning impairment induced by combining these stress treatments. Findings from Experiment 1 indicated that individual differences in learning following RRS + AS were highly dependent on the degree of the left-to-right transition in dopamine-driven activity. In that regard, blocking the receptor unilaterally may have polarized the RRS + AS rats into the two ends of the natural spectrum. Blocking the receptor on the left created a right dominance asymmetry and promoted learning, whereas blocking the receptor on the right created a left dominance asymmetry and impaired learning. This effect appeared to be specific to the medial SNpc. Blocking the receptor in the lateral SNpc of RRS + AS rats, either on the left or the right, neither restored nor further impaired learning. Therefore, although regression analysis in Experiment 1 indicated that a transition from left to right asymmetry in the DLS is also protective in RRS + AS rats, this transition does not appear to have been mediated by CRFR1 in the lateral SNpc.

While the presence of CRFR1 in the SNpc has been previously documented (Sauvage and Steckler, 2001), its role in regulating dopaminergic nigrostriatal projections has not. We found that blocking CRFR1 in either the left or right medial SNpc reduced DA levels in the ipsilateral DMS. However, DOPAC levels were only reduced in the left DMS of AS rats and the right DMS of RRS + AS rats. This is consistent with the left-to-right transition in DA activity we observed in Experiment 1. These findings suggest that activation of CRFR1 in the medial SNpc increases DA availability in the DMS following stress but that local DA release is controlled by other projections or local processes within the DMS. In contrast, blocking CRFR1 in the lateral SNpc increased DA and DOPAC levels in the DLS. This suggests that CRFR1 activation in the medial and lateral SNpc generates opposite effects on DA output into the DMS and DLS, respectively, providing a potential mechanism for regulating the balance between the DMS and DLS following stress.

While CRFR1 is the dominant CRF receptor in the SNpc (Korosi et al., 2006; Beckstead et al., 2009; Schneider et al., 2025), CRFR2 is also present in both neurons and astrocytes in the SNpc (Sharpe et al., 2022) and can influence nigrostriatal projections. To better understand the impact of CRF input into the SNpc and nigrostriatal projections following stress, the role of CRFR2 will also need to be investigated. Furthermore, as CRF is one of the factors regulating CRFR1 expression (Korosi et al., 2006), investigating changes in the CRF input to the SNpc will also be important in future studies. Finally, an important next step will be to identify the specific cell type in which these expression changes occur, as CRF receptors are expressed both on astrocytes and a range of neuron types within the SNpc.

In summary, this study provides new insights into the mechanisms that regulate cognitive flexibility following stress and the role that CRFR1 in the SNpc plays in this process. We suggest that learning is enhanced by CRFR1-driven increases in dopaminergic activity in the left DS following AS, but a left-to-right transition in dopaminergic markers in the DS occurs following a combination of RRS and AS. This transition was partly driven by opposing effects CRFR1 has on the medial and lateral SNpc, potentially reflecting resilience to the effects of stress.

## Synthesis

Reviewing Editor: Michael McDannald, Boston College Decisions are customarily a result of the Reviewing Editor and the peer reviewers coming together and discussing their recommendations until a consensus is reached. When revisions are invited, a fact-based synthesis statement explaining their decision and outlining what is needed to prepare a revision will be listed below. The following reviewer(s) agreed to reveal their identity: NONE. Note: If this manuscript was transferred from JNeurosci and a decision was made to accept the manuscript without peer review, a synthesis may not be available.

In this revised manuscript, Becchi and colleagues uncover lateralized roles for CRFR1 receptors in the in the substantia nigra- impacted by stress - to shape dopamine levels in the medial and lateral dorsal striatum and mediate cognitive flexibility as measured by reinforcer devaluation following reversal. Behaviorally, only repeated restraint stress followed by acute stress impaired reinforcer devaluation following reversal. Activation of nigra/CRF and DS/dopamine in the left hemisphere was associated with resilience to acute stress in naive rats. Yet, this same pattern of activation was associated with stress sensitivity in rats with prior restraint stress experience. Symmetrically, blocking CRCRF1 receptors in the left nigra impaired naive performance in devaluation while facilitating performance following acute stress in restraint experienced rats. The authors positively addressed all concerns from both reviewers and the manuscript is stronger for these edits. I would only note that the authors start the abstract with Chronic restraint stress which is then abbreviated to RRS. I believe they meant to change this to 'Repeated restraint stress.'
