# Supplementary figures and images for: CRF Receptor Type 1 Modulates the Nigrostriatal Dopamine Projection and Facilitates Cognitive Flexibility after Acute and Chronic Stress
Source: eNeuro. 2026 Mar 3;13(3):ENEURO.0019-26.2026. doi: 10.1523/ENEURO.0019-26.2026 (PMC12975668; doi:10.1523/ENEURO.0019-26.2026)

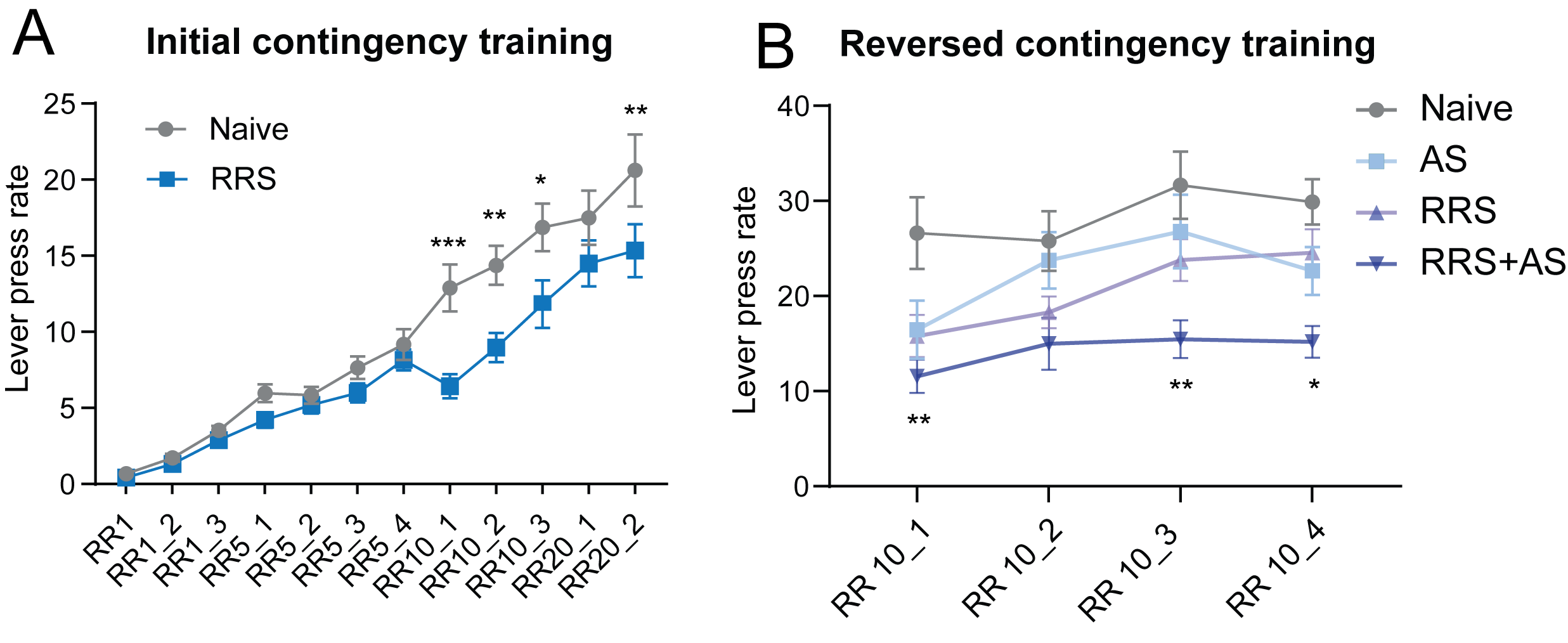

Supplement: Figure 1-1 — Contingency Learning Following Chronic and Acute Stress Exposure (adapted from Mor et al., 2022) A) Group mean lever pressing rates during initial learning. A repeated measures ANOVA showed pressing rates increased over the training, F(11,52) = 30.79, p < .001, but that this increase differed between the CUS and Naïve rats, yielding a stress × training interaction, F(11,52) = 2.29, p = .023. Pressing rates were significantly lower for CUS than Naïve rats, F(1,62) = 6.75, p = .012, suggesting attenuated rigour after CUS. B) Group mean lever pressing rates during reversal learning. A repeated measures ANOVA revealed that pressing rates increased significantly over sessions, F(3,165) = 15.96, p < .001, and there was no interaction between group and sessions, F(9,165) = 1.81, p = .070. There was, however, evidence of a difference in lever pressing between treatment groups F(3,55) = 6.55, p < .001, with main effects for CUS F(1,55) = 12.38, p < .001 as well as AS F(1,55) = 7.42, p = .008. Bonferroni adjusted pairwise comparisons indicated reduced pressing rates in CUS with AS when compared with Naïve rats on training days one (p = .009), two (p = .002) and four (p = .012). Bars represent means ± SEM. *p < .05, **p < .01, ***p < .001. Download Figure 1-1, TIF file. [file eneuro-13-ENEURO.0019-26.2026-s001.tif]

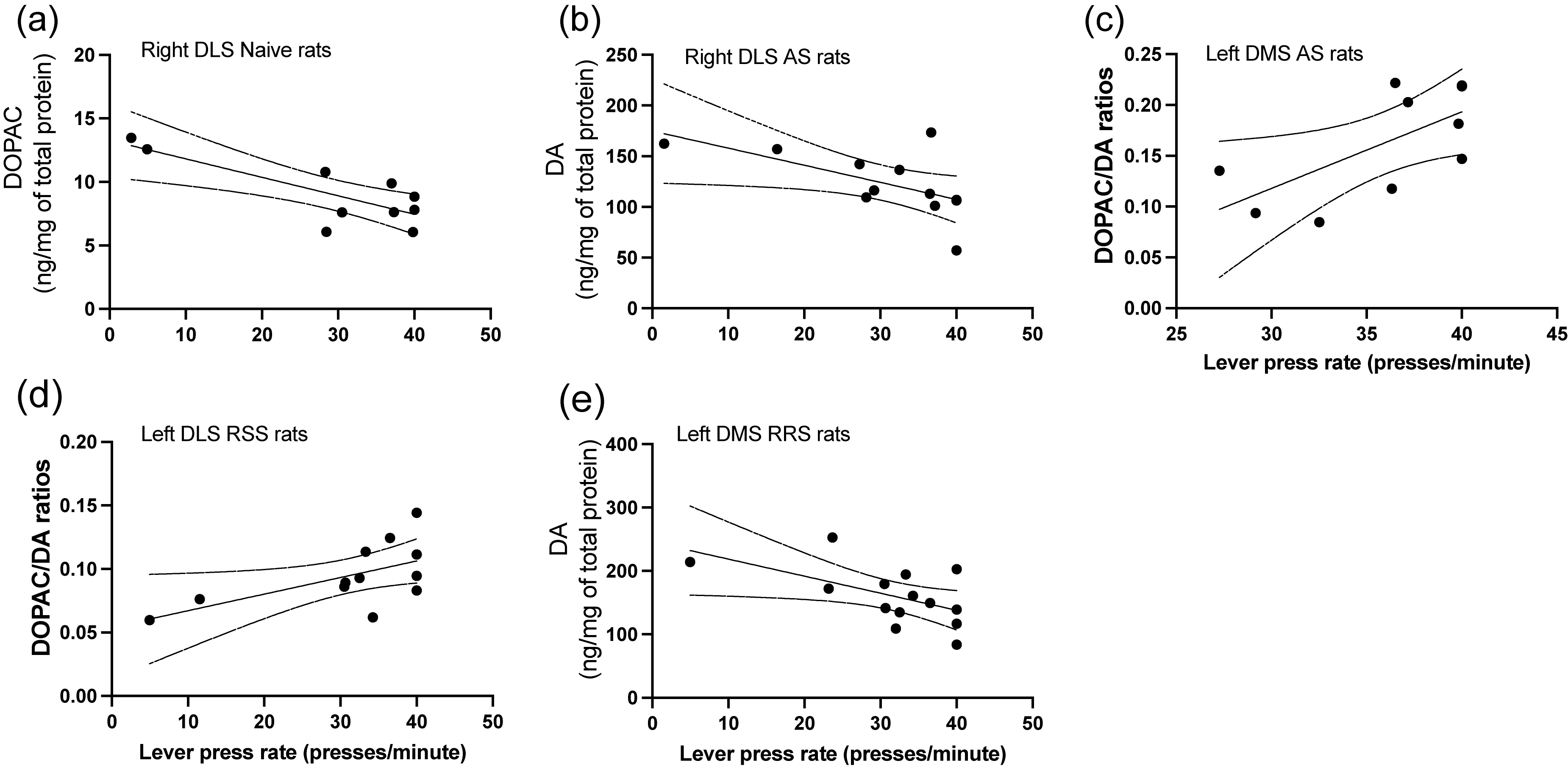

Supplement: Figure 3-1 — Correlations between monoamine levels and behavioral performance during reversal training. During reversal training, pressing rates in both naïve and AS rats decreased with increased dopaminergic activity in the right DLS, with negative correlations with DOPAC in naïve (r2=.627, p = .006) (A) and DA in AS rats (r2=.411, p = .024) (B). In the left DMS, AS and CUS led to opposite associations, with pressing increasing in AS with increased DOPAC/DA ratios (r2=.441, p = .0360) (C) and decreasing with increased DA in CUS rats (r2=.313, p = .037) (D). In contrast to what was found in the left DMS though, CUS rats pressing increased with increased DOPAC/DA in the left DLS (r2=.355, p = .04) (E). Download Figure 3-1, TIF file. [file eneuro-13-ENEURO.0019-26.2026-s003.tif]

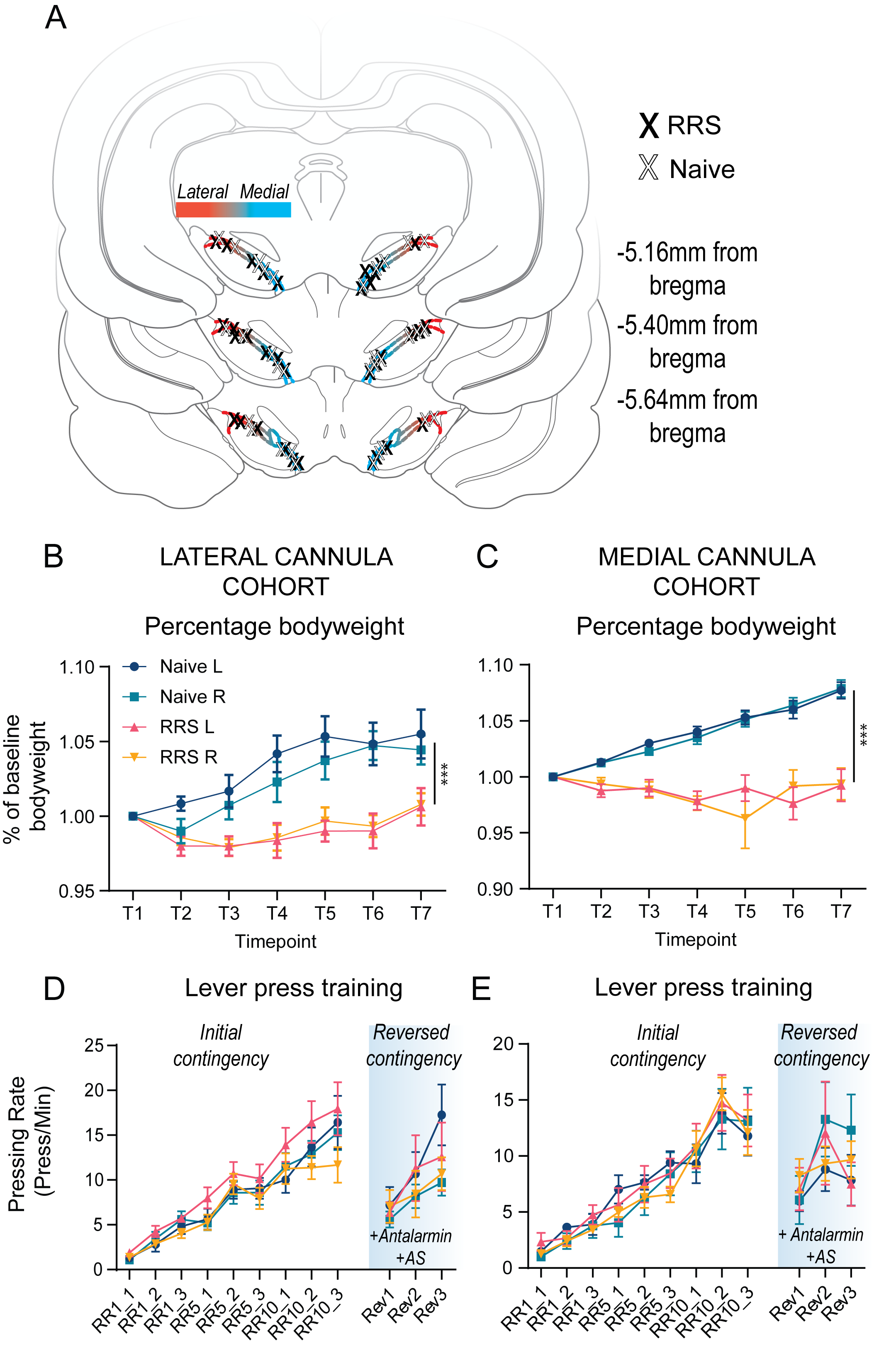

Supplement: Figure 5-1 — The effect of lateralized CRFR1 antagonist infusions on body weight and instrumental training. A) Map of cannula placements for lateral and medial SNpc cohorts. Percentage bodyweight for the lateral cannula cohort (B) and the medial cannula cohort (C) across 2 weeks of CUS or handling. Bodyweight was collected every second day. 2 × 7 ANOVA revealed a significant time x stress interaction, in both the lateral SNpc cohort: F(6, 162) = 8.892, p < .001 and the medial SNpc cohort : F(6, 174) = 13.99, p < .001. D-E) Lever press training for lateral (D) and medial (E) cannula animals during the initial and reversed contingency training. Throughout training, both medial and lateral cannula cohorts quickly acquired lever contingencies, as pressing rates increased over time. During initial contingency training they showed an overall effect of training F(8, 232) = 69.04, p < .001 and F(2.542, 68.64) = 78.10, p < .0001, respectively, with no significant differences between group Naïve and CUS (all Fs < 1) or implantation location, all Fs < 1, p > .05. During training on the reversed contingency, a mixed 2 × 2x3 ANOVA revealed a significant main effect as pressing rate increased over sessions: medial cohort F(2, 58) = 7.963, p < .001, lateral cohort F(1.778, 48) = 23.39, p < .0001. No interactions between session x stressor was found for either cohort: medial infusion F(2, 58) = 1.228, p > .05, lateral infusions F(2, 54) = 1.063, p > .05, neither any interaction between session and infusion side (left or right): medial infusion F(2, 58) = 1.052, p > .05, lateral infusions F(2, 54) = 2.928, p = .062. Bars represent means ± SEM. *** p < .001. Download Figure 5-1, TIF file. [file eneuro-13-ENEURO.0019-26.2026-s004.tif]
