# Supplementary material for: CRF Receptor Type 1 Modulates the Nigrostriatal Dopamine Projection and Facilitates Cognitive Flexibility after Acute and Chronic Stress
Source: eNeuro. 2026 Mar 3;13(3):ENEURO.0019-26.2026. doi: 10.1523/ENEURO.0019-26.2026 (PMC12975668; doi:10.1523/ENEURO.0019-26.2026)
Supplement: Figure 2-1 — Statistical summary table. Tests of within-subjects contrasts and between-subject effects for DA, DOPAC and the DOPAC/DA ratios in DMS and DLS tissue blocks from experiment 1. Download Figure 2-1, DOCX file. [file eneuro-13-ENEURO.0019-26.2026-s002.docx]

|  | | **DMS** | | | | | | | | | | | | | | | |  |  |  |
| --- | --- | --- | --- | --- | --- | --- | --- | --- | --- | --- | --- | --- | --- | --- | --- | --- | --- | --- | --- | --- |
|  |  | Main effect for side | | | | Main effect for AS | | | | | | Main effect for RRS | | | | | |  |  |  |
| DA | | F (1, 44) = 1.315 | P=.258 | | | **F (1,44) = 4.174** | | | **P=.047** | | | F (1,44) = 1.311 | | | P=.258 | | |  |  |  |
| DOPAC | | F (1,44) = 3.352 | P=.074 | | | F (1,44) = .079 | | | P=.78 | | | F (1,44) = 0 | | | P=.989 | | |  |  |  |
| DOPAC/DA | | F (1,41) = .833 | P=.367 | | | **F (1,41) = 9.665** | | | **P=.003** | | | **F (1,41) = 4.295** | | | **P=.045** | | |  |  |  |
|  | | **DLS** | | | | | | | | | | | | | | | |  |  |  |
|  |  | Main effect for side | | | | Main effect for AS | | | | | | Main effect for RRS | | | | | |  |  |  |
| DA | | F (1,40) = .366 | P=.549 | | | F (1,40) = 1.269 | | | P=.267 | | | F (1,40) = .242 | | | P=.626 | | |  |  |  |
| DOPAC | | F (1,40) = 2.141 | P=.151 | | | F (1,40) = .195 | | | P=.661 | | | F (1,40) = 3.048 | | | P=.089 | | |  |  |  |
| DOPAC/DA | | F (1,44) = .96 | P=.758 | | | **F (1,44) = 8.637** | | | **P=.005** | | | F (1,44) = 2.381 | | | P=.13 | | |  |  |  |
|  |  | | | |  |  |  | | |  | | |  | | |  | | |  | |
|  | **DMS** | | | | | | | | | | | | | | | | | | | |
|  | Side*AS*RRS Interaction | | | | | side*AS interaction | | | | | side*RRS interaction | | | | | | AS*RRS interaction | | | |
| **DA** | **F(1,44) = 4.09** | | | **P=.049** | | **F(1,44) = 5.468** | | **P=.024** | | | **F(1,44) = 4.049** | | | **P=0.05** | | | F(1,44) = .03 | | | P.862 |
| DOPAC | **F(1,44) = 5.173** | | | **P=.028** | | F(1,44) = 13.357 | | P=.371 | | | F(1,44) = 2.256 | | | P=0.14 | | | **F(1,44) = 10.047** | | | **P=0.003** |
| DOPAC/DA | F(1,41) = .624 | | | P=.434 | | **F(1,41) = 8.561** | | **P=.006** | | | F(1,41) = 2.723 | | | P=0.107 | | | **F(1,41) = 20.534** | | | **P<.000** |
|  | **DLS** | | | | | | | | | | | | | | | | | | | |
|  | Side*AS*RRS Interaction | | | | | side*AS interaction | | | | side*RRS interaction | | | | | | AS*RRS interaction | | | | |
| DA | F (1,40) = .622 | | | | P=.435 | F(1,40) = 2.058 | P=.159 | | | F(1,40) = .104 | | | P=.749 | | | **F(1,40) = 8.123** | | | **P=.007** | |
| DOPAC | F(1,40) = .226 | | | | P=.637 | F(1,40) = .572 | P=.545 | | | F(1,40) = 1.103 | | | P=.3 | | | **F(1,40) = 14.942** | | | **P<.000** | |
| DOPAC/DA | F(1,44) = .096 | | | | P=.758 | **F(1,44) = 4.68** | **P=.036** | | | F(1,44) = .661 | | | P=.42 | | | F(1,44) = 1.956 | | | P=.169 | |

**Figure 2-1: Statistical summary table.** Tests of within-subjects contrasts and between-subject effects for DA, DOPAC and the DOPAC/DA ratios in DMS and DLS tissue blocks from experiment 1
