# Supplementary material for: CRF Receptor Type 1 Modulates the Nigrostriatal Dopamine Projection and Facilitates Cognitive Flexibility after Acute and Chronic Stress
Source: eNeuro. 2026 Mar 3;13(3):ENEURO.0019-26.2026. doi: 10.1523/ENEURO.0019-26.2026 (PMC12975668; doi:10.1523/ENEURO.0019-26.2026)
Supplement: Figure 6-1 — Statistical summary table. Tests of within-subjects contrasts and between-subject effects for DA and DOPAC in the DMS and DLS tissue blocks from Experiment 2. Download Figure 6-1, DOCX file. [file eneuro-13-ENEURO.0019-26.2026-s005.docx]

|  | **DMS** | | | | | | |
| --- | --- | --- | --- | --- | --- | --- | --- |
|  | Main effect for Infusion | | Main effect for RRS | | Infsion*RRS interaction | | |
| DA left infusion | **F (1, 23) = 30.14** | **P<0.0001** | **F (1, 23) = 5.168** | **P=0.0327** | F (1, 23) = 0.4887 | | P=0.4915 |
| DA right infusion | **F (1, 24) = 30.89** | **P<0.0001** | F (1, 24) = 0.05814 | P=0.8115 | F (1, 24) = 0.3807 | | P=0.5430 |
| DOPAC left infusion | **F (1, 23) = 18.48** | **P=0.0003** | **F (1, 23) = 8.620** | **P=0.0074** | F (1, 23) = 2.400 | | P=0.1350 |
| DOPAC right infusion | **F (1, 24) = 6.165** | **P=0.0204** | F (1, 24) = 0.1614 | P=0.6914 | F (1, 24) = 2.342 | | P=0.1390 |
|  | **DLS** | | | | | | |
|  | Main effect for Infusion | | Main effect for RRS | | Infsion*RRS interaction | | |
| DA left infusion | F (1, 26) = 2.389 | P=0.1343 | F (1, 26) = 0.01231 | P=0.9125 | **F (1, 26) = 5.374** | **P=0.0286** | |
| DA right infusion | **F (1, 24) = 9.657** | **P=0.0048** | F (1, 24) = 0.2919 | P=0.5940 | F (1, 24) = 3.555 | P=0.0715 | |
| DOPAC left infusion | **F (1, 25) = 4.853** | **P=0.0370** | F (1, 25) = 0.005187 | P=0.9432 | F (1, 25) = 2.905 | P=0.1007 | |
| DOPAC right infusion | **F (1, 24) = 10.36** | **P=0.0037** | F (1, 24) = 0.1791 | P=0.6760 | F (1, 24) = 3.379 | P=0.0785 | |

**Figure 6-1: Statistical summary table**: Tests of within-subjects contrasts and between-subject effects for DA and DOPAC in the DMS and DLS tissue blocks from Experiment 2**.**
